# Failure Analysis of Cracking of Cast Aluminum Alloy Manhole Cover

**DOI:** 10.3390/ma16041561

**Published:** 2023-02-13

**Authors:** Facai Ren, Hezong Li

**Affiliations:** 1 Shanghai Institute of Special Equipment Inspection and Technical Research, Shanghai 200062, China; 2 Key Laboratory of Intelligent Industrial Equipment Technology of Hebei Province, Hebei University of Engineering, Handan 056038, China

**Keywords:** crack failure, cast aluminum alloy, manhole cover

## Abstract

In this paper, the abnormal fracture failure of a ZL104 aluminum alloy quick-opening manhole cover of a cement tank truck is systematically studied to discover the root cause of an accident. The unloading operation procedures of cement tank trucks, the effectiveness of safety valves, the chemical composition, mechanical properties and material quality of aluminum alloy manhole covers, and the macroscopic and microscopic morphology of fractures were comprehensively analyzed. The results show that although the Mg content in the chemical composition of an aluminum alloy manhole cover exceeds the standard, it is not the root cause of the accident. The root cause of the failure is that, during the unloading operation, the operator did not strictly follow the unloading procedures. One of the buckles was in the released state, which led to uplift cracking, resulting in the successive cracking and slipping of adjacent buckles, and the manhole cover finally cracked and flew out. Based on the failure causes, suggestions are put forward to prevent the manhole cover from failing during the unloading operation of cement tank trucks in the future.

## 1. Introduction

Aluminum alloys are generally divided into cast aluminum alloys and wrought aluminum alloys. The main difference between the two is the different forming methods. In addition to the general characteristics of aluminum, aluminum alloys have some specific characteristics which are due to the different types and quantities of the alloying elements that are added. Aluminum alloy has high strength, good casting and plastic processing properties, good electrical and thermal conductivity, good corrosion resistance and weldability [1,2,3]. Aluminum alloys can be used as structural materials and are widely used in aerospace, aviation, transportation, construction, electromechanical and daily necessities.

In view of the wide application of aluminum alloys, their properties have been thoroughly investigated, including the effects of alloy element addition [4,5], deformation process [6,7], welding parameters [8,9] and aging treatment [10,11,12,13] on the microstructure, mechanical properties and corrosion resistance. At the same time, other scholars have studied the failure behavior of aluminum alloy parts. Luiz et al. [14] studied the premature corrosion of a power connector made of silver-plated ASTM 356 cast aluminum alloy in substations. Defects formed in the electroplating process expose the aluminum substrate and silver coating in the electrolyte, resulting in severe galvanic corrosion of the substrate. Li et al. [15] studied two aircraft frame components made of aviation material ZL205A and found the cause of fatigue cracks to be casting porosity defects on the outer surface of the frame. Liu et al. [16] conducted a failure analysis on an aluminum alloy drill pipe and studied the causes of pits and parallel transverse cracks. They found that the main reasons for the failure are the brittleness sensitivity of the intermittent banded second phase and the sensitivity of inclusions to corrosive mud environments. Reinke et al. [17] studied the fatigue performance of wires drawn from two cables made of 6201 aluminum alloy and found that low heat-treatment efficiency and existing iron impurities that may come from the raw materials used in the manufacturing process will shorten their fatigue life. Carboni [18] studied the premature failure of an aluminum alloy ultrasonic welding electrode made of AA7075-T6 and found that segregation is the main reason for crack initiation.

This paper analyzes the failure of a quick-opening aluminum alloy manhole cover of a cement tank truck. The analysis process includes the unloading procedures, the effectiveness of the safety valve, the chemical composition and mechanical property inspection of the base material, and the macroscopic and microscopic inspection of the fracture area. According to the analysis results, the root cause of the manhole cover’s failure is determined, and corresponding countermeasures are proposed.

## 2. Description of Manhole Cover Fracture Failure

**Figure 1:**
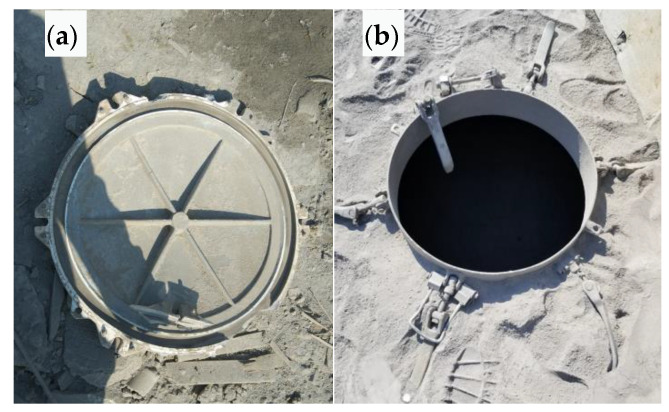
Manhole cover and manhole ring: (**a**) broken cover, (**b**) manhole ring.

During the unloading of a cement tank truck transferring cement dust to a storage tank under pressure, one manhole cover ruptured and flew approximately 20 m away, while the other manhole cover was intact. The cracked manhole cover and its corresponding manhole ring are shown in Figure 1. The safety buckle of the manhole cover installed on the tank body has been straightened and no obvious deformation or other failure is observed on the other parts, as shown in Figure 1b.

## 3. Materials and Experimental Methods

### 3.1. Materials

After checking the relevant quality certificates of the manhole cover, it was determined that it is made of cast aluminum alloy ZL104. The material contained in the tank is cement dust. Through careful inspection of the accident site, no corrosive medium or corrosion phenomenon was found.

### 3.2. Safety Valve Effectiveness

The design pressure of the tank body of the cement tanker is 0.4 MPa, the rated pressure of the manhole cover is 0.25 MPa, and the opening pressure of the safety valve is 0.22 MPa. According to the operation manual, when the pressure in the tank reaches about 0.2 MPa, one should open the blowing valve, clean the feeding pipe, open the discharge butterfly valve of the front and rear silos, and start discharging. After testing, when the pressure exceeds 0.22 MPa, the safety valve can effectively expand to relieve pressure. Therefore, the idea that the manhole cover failed due to the high pressure in the cement tank truck caused by the failure of the safety valve can be ruled out.

### 3.3. Experimental Methods

The quality and fracture of the manhole cover body were analyzed macroscopically and microscopically to determine the root cause of manhole cover fracture failure. The preparation process of the metallographic sample was to cut a sample from the manhole cover, and then polish and etch it (5% hydrofluoric acid aqueous solution, guaranteed reagent). We used an optical microscope for inspection. When grading the pinhole, the sample was etched with 15% sodium hydroxide aqueous solution (analytical reagent). The chemical composition of the manhole cover was detected by optical emission spectrometry.

The test and inspection is primarily based on: *General Rules for Analytical Scanning Electron Microscopy* (JY/T 010-1996) [19]; *Optical Emission Spectrometric Analysis Method of Aluminum and Aluminum Alloys* (GB/T 7999-2015) [20]; *Inspection Methods of Microstructure for Metals* (GB/T 13298-2015) [21]; *Metallograph of Cast Aluminum Alloys Cast Aluminum–Silicon Alloys Modification* (JB/T 7946.1-1999) [22]; *Metallic Materials—Brinell Hardness Test Part 1: Test Method* (GB/T 231.1-2009) [23]; etc.

## 4. Results and Discussion

### 4.1. Macro Morphology Analysis

#### 4.1.1. Overall Macro Morphology

**Figure 2:**
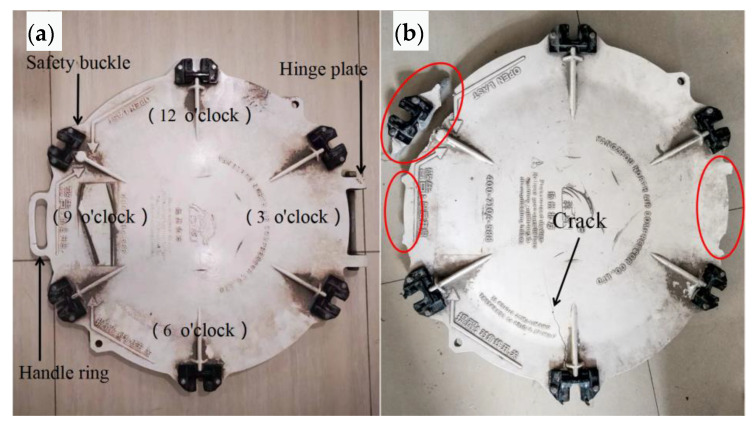
Macro morphology of manhole covers: (**a**) intact, (**b**) cracked.

The macro morphology of intact and cracked manhole covers is shown in Figure 2. The shape of the quick-opening manhole cover of the cement tank truck is shown in Figure 2a. The nine o’clock position is equipped with a pull ring, and the three o’clock position is equipped with a hinged plate. The manhole cover is uniformly distributed with six handles for sealing (equipped with black engineering plastic pressure block), among which there is a buckle at the ten o’clock position (released last when opening). The outer diameter of the quick-opening manhole cover is about 600 mm, and the middle wall thickness of the manhole cover is about 8 mm. The nominal maximum allowable pressure is 0.25 MPa. The material is ZL104, which is molded by casting. The model of the manhole cover is TY600 × 520. The morphology of the cracked manhole cover is shown in Figure 2b. The handle ring at the nine o’clock position and the hinged plate at the three o’clock position had fallen off. Radial cracks can be seen in the sealing buckle area at the six o’clock position. The safety buckle was broken and separated at the ten o’clock position.

**Figure 3:**
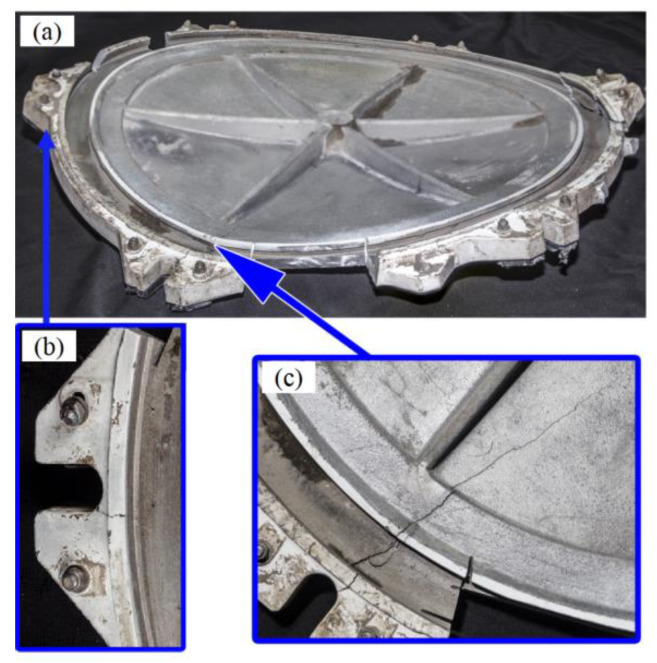
Deformation and fracture morphology of manhole cover: (**a**) overall morphology, (**b**) crack morphology at four o’clock, (**c**) crack morphology at six o’clock.

The morphology observed from the inner surface is shown in Figure 3. The six reinforcing ribs on the inner surface correspond to the six sealing buckles. There is an abnormal bulge in the sealing buckle area at six o’clock. The uplift clearance is about 68 mm, which is deformed symmetrically along the radial direction, accompanied by cracks with radial distribution, and the length is about 205mm. From the external and internal surface, the crack runs through the wall thickness, as shown in Figure 3c. Cracks originating from the inner surface can be seen at the corners of the sealing buckle at four o’clock and the sealing buckle at eight o’clock on both sides of the crack. The two cracks are intended to be related to the uplift at six o’clock, as shown in Figure 3b.

#### 4.1.2. Cracked Surface at the Six O’Clock Position

**Figure 4:**
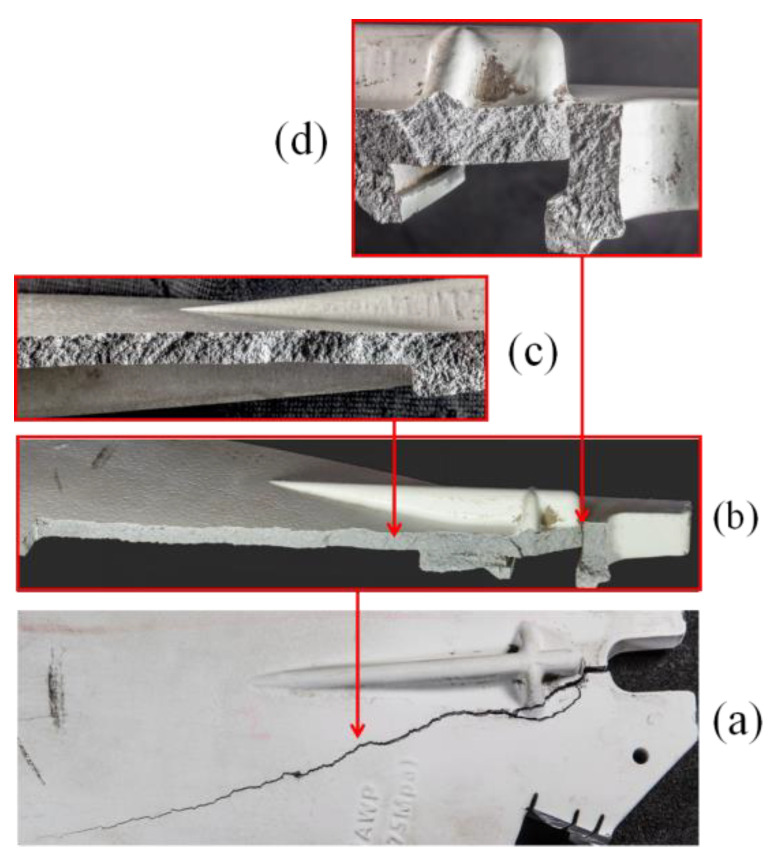
Macro morphology of crack and crack surface at six o’clock: (**a**) whole crack, (**b**) crack after opening, and (**c**,**d**) two parts of the crack surface.

The crack morphology at the six o’clock position is shown in Figure 4a. One end of the crack is in the groove area of the seal buckle, the opening is wide, and the other end is thin, indicating that the crack starts at the outer circle groove and develops around the stiffener, with an extension length of about two-thirds of the radius. The macro morphology after crack opening is as shown in Figure 4b. No obvious metallurgical defects are found on the crack surface, the crack surface is relatively rough, and no oxidation phenomenon is found. Close observation reveals oblique spreading stripes on the crack surface, as shown in Figure 4c, indicating that the crack initially propagates towards the direction of the outer circle. It can be further seen that the crack starts at the corner of the outer surface, as shown in Figure 4d.

#### 4.1.3. Broken Area of Safety Buckle

**Figure 5:**
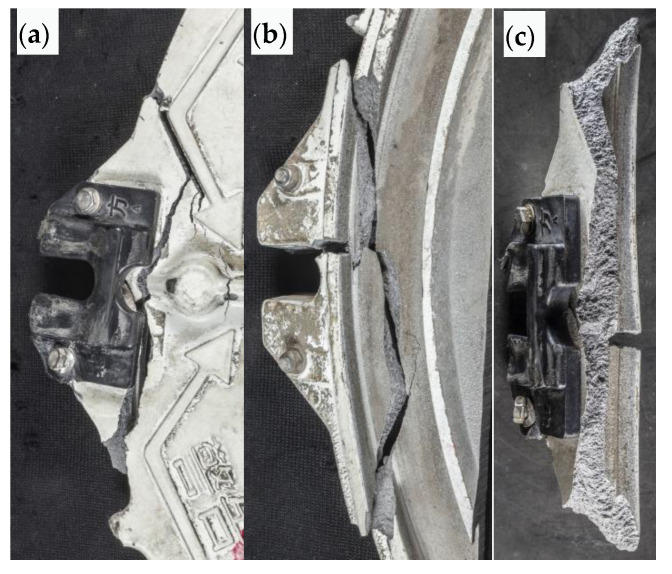
Macro morphology of safety buckle fracture surface: (**a**,**b**) external surface and internal cavity surface morphology, respectively, of cracked block after assembly, (**c**) macromorphology of crack surface.

The appearance of the outer surface of the split area of the safety buckle after splicing is shown in Figure 5a. The cracks are distributed along the tangential direction of the compression block and distributed along the corner of the protruding reinforcement in the local area on the left. From the inner cavity surface, as shown in Figure 5b, there is a short crack at the corner of the buckle groove that intersects with the tangential main crack, forming a T shape. The main crack turns at the intersection, indicating that the main crack is not formed by primary expansion. The macro morphology of the fracture surface is shown in Figure 5c. The top of the figure shows the inner cavity surface. No obvious metallurgical defects or oxidation phenomena are found on the fracture surface. The pattern of the fracture surface is rough. Its trend indicates that the crack mainly extends from the inner cavity to the outer surface, starting from the outer circles on both sides, and intersecting at the center.

#### 4.1.4. Fracture Surface of Hinged Plate

**Figure 6:**
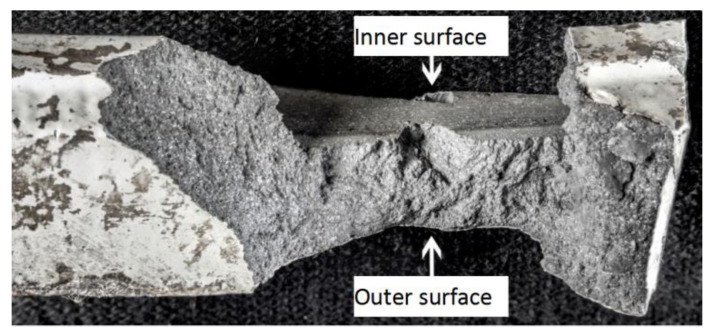
Fracture surface morphology of the hinged plate.

The fracture surface morphology of the hinged plate is shown in Figure 6. The top of the figure shows the inner cavity surface. The fracture surface is relatively rough, and no obvious metallurgical defects or oxidation phenomena are found. Extended stripes can be seen on the fracture surface from the outer to the inner surface.

### 4.2. SEM Analysis of Fracture Surface

#### 4.2.1. Cracked Surface at the Six O’Clock Position

**Figure 7:**
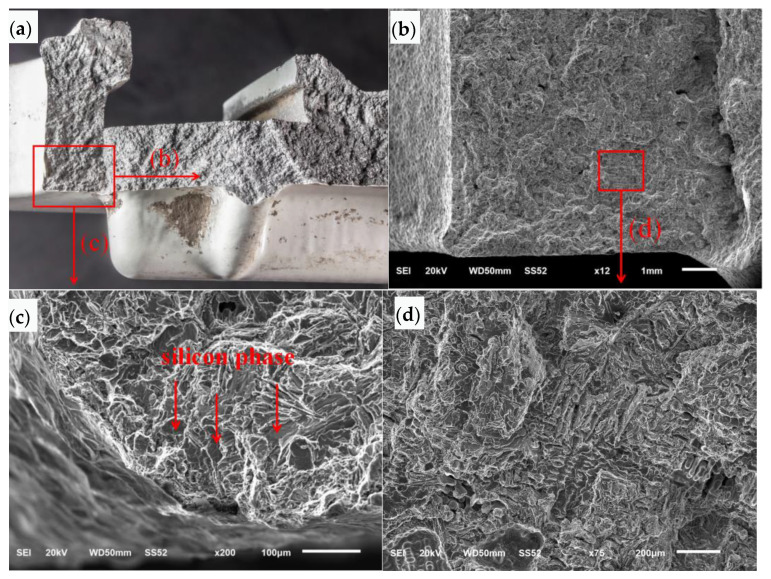
SEM morphology of crack initiation zone: (**a**) fracture surface, (**b**) low magnification, (**c**) high magnification, (**d**) local area of the crack surface.

The fracture surface at the 6 o’clock position was analyzed by SEM as shown in Figure 7a. The SEM morphology of the crack initiation area at the six o’clock position is shown at low magnification in Figure 7b. The left and bottom sides of the figure are the outer surface. The crack surface extends diagonally inward from the outer surface and the corner area of the outer surface. The SEM morphology at high magnification is shown in Figure 7c. The fracture surface is cleaved and faceted along the silicon phase. The SEM morphology in the local area of the crack surface is shown in Figure 7d. There are distributed loose defects, and the crack surface in this area is of a cellular dendrite morphology, showing free surface morphology.

**Figure 8:**
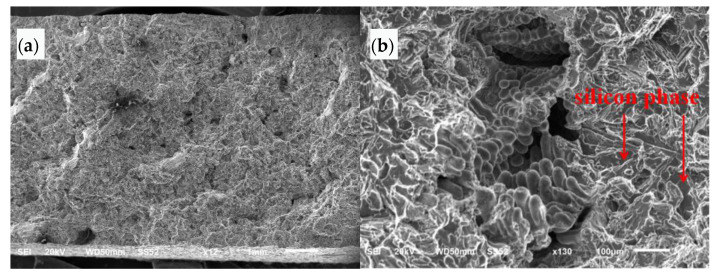
SEM morphology of crack propagation zone: (**a**) low magnification, (**b**) high magnification.

The SEM morphology of the crack propagation area is shown at low magnification in Figure 8a. The crack surface is undulated and extended stripes can be seen from the bottom left to top right. The SEM morphology of the crack propagation area at high magnification is shown in Figure 8b. The crack surface is cleaved and faceted along the silicon phase. Porous defects can be seen in local areas, and cellular dendritic morphology can be seen around the loose area, showing free surface morphology.

#### 4.2.2. Fracture Area of Safety Buckle

**Figure 9:**
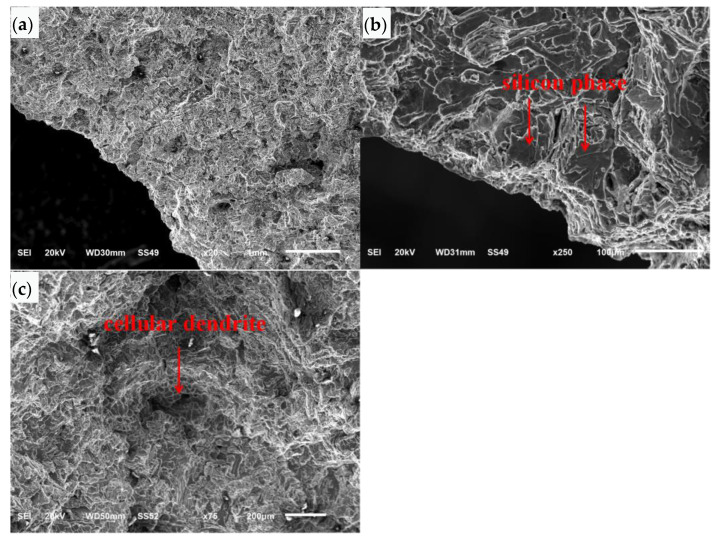
SEM morphology of initiation zone and propagation zone of the safety buckle: (**a**) low magnification, (**b**) high magnification, (**c**) section expansion area.

The SEM morphology of the initial area of the safety buckle fracture section is shown at low magnification in Figure 9a. The bottom left side of the figure is the surface of the inner cavity, and the top right side is the section. The section is relatively rough, and extended stripes can be seen from the beginning of the inner cavity to the outside. The SEM morphology of the initial area of the safety buckle fracture section is shown at high magnification in Figure 9b. The fracture surface is cleaved and faceted along the silicon phase. The SEM morphology of the section expansion area is shown in Figure 9c. The morphology is cleaved and faceted along the silicon phase, loose defects can be seen in local areas, and cellular dendritic morphology can be seen in this area.

#### 4.2.3. Fracture Area of Hinged Plate

**Figure 10:**
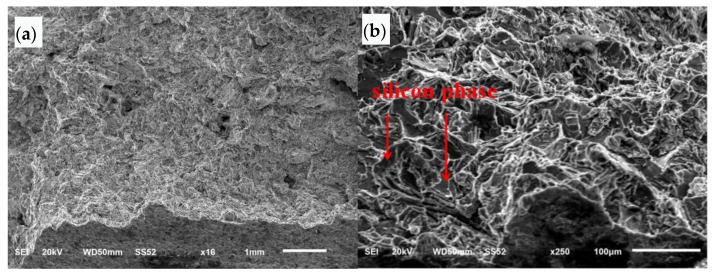
SEM morphology of the fracture initiation zone of the hinged plate: (**a**) low magnification, (**b**) high magnification.

The SEM morphology of the initial area of the hinged plate section is shown at low magnification in Figure 10a. The bottom left side of the figure is the outer surface, and the top side of the figure is the section. The section is relatively rough, and extended stripes can be seen from the beginning to the top. The SEM morphology at high magnification is shown in Figure 10b. The cross section is cleaved and faceted along the silicon phase.

**Figure 11:**
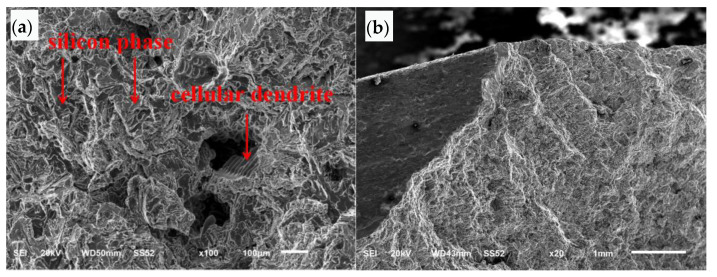
SEM morphology of the hinged plate: (**a**) fracture propagation region, (**b**) final fracture region.

The SEM morphology of the fracture propagation area of the hinged plate is shown in Figure 11a. The morphology is cleaved and faceted along the silicon phase. Loose defects can be seen in local areas, and the section of this area is cellular dendrite. The SEM morphology of the final fracture zone of the hinged plate is shown in Figure 11b, which shows tear morphology.

### 4.3. Microstructure Analysis

#### 4.3.1. Normal Section of Crack Surface at the Six O’Clock Position

**Figure 12:**
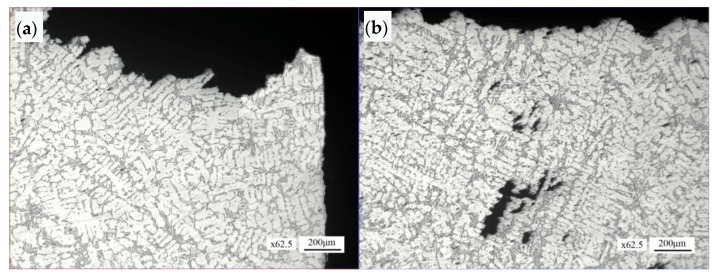
Microstructure morphology of the normal section of the crack surface at six o’clock: (**a**) fracture initiation region, (**b**) fracture propagation region.

The microstructure distribution morphology of the initial area of the crack surface at the six o’clock position is shown in Figure 12a. The right side of the figure is the outer circular surface, and the top side of the figure is the crack surface. The crack surface fluctuates. The microstructure of this area is α (Al) + Si phase. In the fracture surface propagation area, the fracture surface fluctuates greatly, multiple loose pores and pores can be seen in the sub surface area. The organization in local areas is dendritic, as shown in Figure 12b.

#### 4.3.2. Normal Section of Safety Buckle Fracture Section

**Figure 13:**
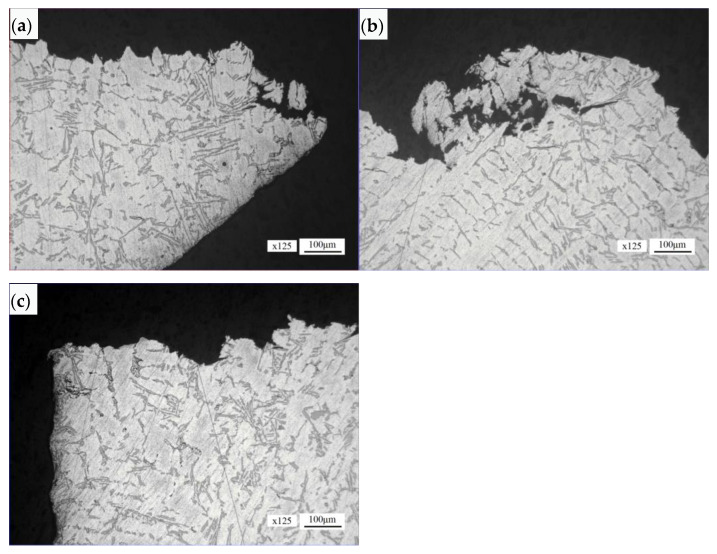
Microstructure morphology of the normal section of the safety buckle fracture section: (**a**) fracture initiation region, (**b**) fracture propagation region, (**c**) final fracture region.

The microstructure morphology of the initial area of the safety buckle fracture section is shown in Figure 13a. The section fluctuates greatly. The microstructure morphology of the section propagation area is shown in Figure 13b. The section is relatively undulated, and loose defects can be seen in local areas around the section, with the loose size of about 0.35 mm × 0.16 mm. The microstructure morphology of the final fracture area is shown in Figure 13c. The microstructure of the initial area, propagation area and final fracture of the safety buckle fracture section is α (Al) + Si phase.

#### 4.3.3. Microstructure of Manhole Cover Matrix

**Figure 14:**
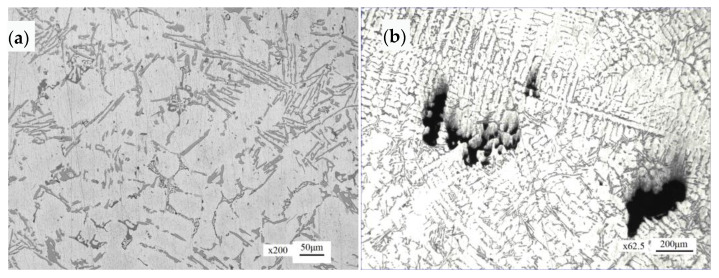
Microstructure morphology: (**a**) manhole cover matrix, (**b**) porous casting defects.

The microstructure of the manhole cover matrix is α (Al) + Si phase, with dendritic distribution in local areas, as shown in Figure 14a. In some areas, loose casting defects can be seen, and the loose size is about 0.42 mm × 0.08 mm, as shown in Figure 14b.

#### 4.3.4. Morphology of the Normal Section near the Outer Circle

**Figure 15:**
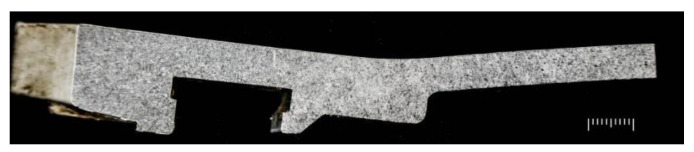
Morphology of the normal section near the outer circle.

Low magnification inspection of the normal section near the outer circle of the manhole cover rated the pinhole degree as Grade 3 (the highest level is Grade 5), as shown in Figure 15.

### 4.4. Mechanical Property Analysis

According to Metallic materials—Tensile testing Part 1: Method of test at room temperature (GB/T 228.1-2010), three specimens should be taken from the near outer circle area of the failed manhole cover for tensile testing. The diameter of the specimen is 8mm. The average value was taken from three repeat tests and the results are shown in Table 1. In the table, F represents “as cast”, J represents “metal mold casting”, T1 represents “artificial aging”, and T6 represents “solution treatment plus complete artificial aging”. It can be seen from the tensile test results that the tensile strength and elongation after fracture of the sample meet the technical requirements of as-cast or metal mold casting after artificial aging heat treatment.

According to *Metallic Materials—Brinell Hardness Test Part 1: Test Method* (GB/T 231.1-2009), Brinell hardness was measured on the manhole cover substrate. The results of three measurements are shown in Table 2. It can be seen that the hardness of the sample meets the requirements of *Casting Aluminum Alloy* (GB/T 1173-2013).

### 4.5. Chemical Composition Analysis

The matrix of the manhole cover was sampled and analyzed for chemical composition. The results are shown in Table 3. According to the chemical analysis results, the content of Mg in the manhole cover is 0.45%, which exceeds the requirement of 0.17~0.35 for Mg content in ZL104 per the relevant standard outlined in *Casting Aluminum Alloy* (GB/T 1173-2013). Mg is one of the most used alloying elements. In Al–Si–Mg alloy, the strengthening effect is mainly produced by precipitation of Mg_2_Si phase [24]; however, the content of Mg should be strictly controlled. The presence of Mg in Al–Si alloy will cause the decrease of liquidus temperature and eutectic point temperature. The decrease of eutectic point temperature will slow down the solidification rate and lead to the coarsening of eutectic Si phase, thus reducing the performance of the alloy [25].

## 5. Conclusions and Proposals

According to the above failure analysis, the main conclusions are as follows:

(1) The porosity and pinhole of the manhole cover meet the standard requirements, so the fracture was not caused by material quality problems.

(2) During the unloading process, one of the buckles was in an abnormal release state, which was the root cause of the manhole cover failure.

(3) With one bulge cracking, the adjacent buckles cracked and slipped, and finally other parts broke instantaneously due to mandatory constraints.

With reference to the above failure analysis conclusions, the following proposals are proposed:

(1) The unloading process of cement tank vehicles should be strictly followed. Before unloading, one check whether the manhole cover is tightly pressed and whether the safety buckle is in a locked state.

(2) One should install the quick opening interlock device to prevent accident caused by human error.

## Figures and Tables

**Table 1:** Tensile test results of manhole cover base.

Specimen		Tensile Strength (N/mm^2^)	Elongation (%)
No. 1		155	2.42
No. 2		162	4.15
No. 3		154	3.00
Average		157	3.19
ZL104 (GB/T 1173-2013)	F	≥150 × 75% = 112.5	≥2 × 50% = 1
	J, T1	≥200 × 75% = 150	≥1.5 × 50% = 0.75
	J, T6	≥240 × 75% = 180	≥2 × 50% = 1

**Table 2:** Brinell hardness of manhole cover base.

Specimen	Brinell Hardness (HBW)		
Manhole cover	64.0	63.0	64.0
ZL104 (GB/T 1173-2013)	≥50		

**Table 3:** Chemical composition of manhole cover.

Element	Si	Mg	Mn	Fe	Zn	Ti	Sn	Cu	Pb	Ti + Zr
Manhole cover	8.81	0.45	0.24	0.22	0.005	0.032	0.009	0.030	0.007	0.032
ZL104 (GB/T1173-2013)	8.0~10.5	0.17~0.35	0.2~0.5	≤0.9	≤0.25	≤0.2	≤0.05	≤0.1	≤0.05	≤0.15

## Data Availability

Data available on request.

## References

[1] Liu M. (2021). Comparative study on corrosion resistance of Marine 5083 and 5059 aluminum alloy slabs. Ship Sci. Technol..

[2] Rambabu P.P.N.K.V., Eswara Prasad N., Kutumbarao V.V., Wanhill R.J.H. (2017). Aluminium alloys for aerospace applications. Aerosp. Mater. Mater. Technol..

[3] Derry C., Robson J. (2008). Characterisation and modelling of toughness in 6013-T6 aerospace aluminium alloy friction stir welds. Mater. Sci. Eng. A.

[4] Sunde J., Marioara C., Holmestad R. (2020). The effect of low Cu additions on precipitate crystal structures in overaged Al-Mg-Si(-Cu) alloys. Mater. Charact..

[5] Mochugovskiy A., Kotov A., Ghayoumabadi M.E., Yakovtseva O., Mikhaylovskaya A. (2021). A high-strain-rate superplasticity of the Al-Mg-Si-Zr-Sc alloy with Ni addition. Materials.

[6] Wang D., Zhang W., Huang S., Yi Y., He H. (2022). Effect of three-dimensional deformation at different temperatures on microstructure, strength, fracture toughness and corrosion resistance of 7A85 aluminum alloy. J. Alloy. Compd..

[7] Wang D., Yi Y., Li C., Huang S., He H., Zhang J. (2021). Effects of different multidirectional forging processes on the microstructure and three-dimensional mechanical properties of ultra-high strength aluminum alloys. Mater. Sci. Eng. A.

[8] Kim Y., Fujii H., Tsumura T., Komazaki T., Nakata K. (2006). Effect of welding parameters on microstructure in the stir zone of FSW joints of aluminum die casting alloy. Mater. Lett..

[9] Xu T., Zhu Z., Mi G., Wang L., Li M., Ma X. (2022). Revealing the mechanism of laser welded aluminum alloy joints in-situ reinforced by carbon nanotubes based on microstructural evolution. Mater. Charact..

[10] Lei G., Wang B., Lu J., Wang C., Li Y., Luo F. (2022). Microstructure, mechanical properties, and corrosion resistance of continuous heating aging 6013 aluminum alloy. J. Mater. Res. Technol..

[11] Kairy S., Rometsch P., Davies C., Birbilis N. (2017). On the intergranular corrosion and hardness evolution of 6xxx series Al alloys as a function of Si: Mg ratio, Cu content, and aging condition. Corrosion.

[12] Li Y., Qin W., Yu S., La J., Fu Y., Li J., Yang W., Zhan Y. (2021). Effect of aging treatment on the corrosion resistance properties of 7N01 extrusion aluminum alloy. Materials.

[13] Zhan X., Tang J., Li H., Liang X., Lu Y., Che Y., Tu W., Zhang Y. (2020). Effects of non-isothermal aging on mechanical properties, corrosion behavior and microstructures of Al-Cu-Mg-Si alloy. J. Alloy. Compd..

[14] Luiz L., Gobi C., Andrade J., Peres E., Paro O. (2022). Failure analysis of corroded 500 kV connectors made of silver plated ASTM 356 aluminum alloy. Eng. Fail. Anal..

[15] Li B., Shen Y., Hu W. (2011). Casting defects induced fatigue damage in aircraft frames of ZL205A aluminum alloy—A failure analysis. Mater. Des..

[16] Liu W., Li J., Zhong Y., Shi T., Zhang J., Li S. (2022). Failure analysis on aluminum alloy drill pipe with pits and parallel transverse cracks. Eng. Fail. Anal..

[17] Reinke G., Badibanga R., Pestana M., Ferreira J., Araujo J., Silva C. (2020). Failure analysis of aluminum wires in all aluminum alloy conductors -AAAC. Eng. Fail. Anal..

[18] Carboni M. (2014). Failure analysis of two aluminium alloy sonotrodes for ultrasonic plastic welding. Int. J. Fatigue.

[19] (1996). General Rules for Analytical Scanning Electron Microscopy.

[20] (2015). Optical Emission Spectrometric Analysis Method of Aluminum and Aluminum Alloys.

[21] (2015). Inspection Methods of Microstructure for Metals.

[22] (1999). Metallograph of Cast Aluminium Alloys Cast Aluminium-Silicon Alloys-Modification.

[23] (2009). Metallic Materials-Brinell Hardness Test-Part 1, Test Method.

[24] Cao F., Li Z., Zhang N., Ding H., Yu F., Zuo L. (2013). Superplasticity, flow and fracture mechanism in an Al-12.7Si-0.7Mg alloy. Mater. Sci. Eng. A.

[25] Yıldırım M., Özyürek D. (2013). The effects of Mg amount on the microstructure and mechanical properties of Al-Si-Mg alloys. Mater. Des..

